# The Morel-Lavallée Lesion as a Rare Differential Diagnosis for Recalcitrant Bursitis of the Knee: Case Report and Literature Review

**DOI:** 10.1155/2012/593193

**Published:** 2012-12-20

**Authors:** Ivor S. Vanhegan, B. Dala-Ali, L. Verhelst, P. Mallucci, Fares S. Haddad

**Affiliations:** ^1^Department of Orthopaedics, University College Hospital, 235 Euston Road, London NW1 2BU, UK; ^2^Department of Plastic Surgery, Royal Free Hospital, Pond Street, London NW3 2QG, UK

## Abstract

A 72 year-old-male was referred to our institution with recalcitrant prepatellar bursitis. The injury was sustained after striking his right knee against a post whilst horse riding 9 months ago. Previous treatments included repeated aspiration and excision of the bursa with elastic compression bandaging. A diagnosis of a Morel-Lavallée internal degloving injury was made, and the lesion was satisfactorily managed by an internal quilting procedure to eliminate the potential dead space. A review of the literature reveals 29 published reports of Morel-Lavallée lesions with sufficient information for inclusion. These came from 14 separate countries with a total of 204 lesions in 195 patients. The most common anatomical location was the greater trochanter/hip (36%), followed by the thigh (24%) and the pelvis (19%). Most were managed surgically with evacuation of the haematoma and necrotic tissue followed by debridement, which was often repeated (36%). Conservative treatment with percutaneous aspiration and compression bandaging was the next most common treatment (23%). The knee was the fourth most common region affected (16%), and only 3 other lesions in the literature have been managed with a quilting procedure.

## 1. Introduction

In 1853 Maurice Morel-Lavallée first described the closed degloving lesion with which he became eponymous. Frequently resulting from blunt shearing or tangential forces, the hypodermis becomes separated away from the underlying fascia [1]. The space that is created becomes filled with blood, lymph, and necrotic fat around which granulation tissue may become organised into a fibrous pseudocapsule. The presence of such a capsule prevents absorption of the contained fluid and explains why clinical presentation of these lesions can be several months or even years following injury.

Letournel and Judet were the first to use the name “Morel-Lavallée lesion” (MLL) in their classification of acetabular fractures. In this context, the lesion referred specifically to the closed degloving injuries over the region of the greater trochanter [2]. There is an increased likelihood of underlying pelvic and acetabular fractures where these lesions are encountered [3, 4] and their presence is associated with increased surgical site infection rates [5, 6]. It is now accepted that these collections can be encountered at multiple locations in the body: head [7], abdominal wall [8, 9], pelvis [10], lumbosacral region [11, 12], gluteal [13, 14], thigh [15, 16], knee [7, 17], and calf [18].

This paper highlights an unusual presentation of persistent knee swelling and addresses the literature regarding diagnosis, management, and outcome of MLL. 

## 2. Case Report

A fit 72-year-old gentleman was referred to our institution with a 9-month history of prepatellar swelling. The swelling was reported to have arisen 3 days after striking his right knee against a post whilst horse riding. The gentleman was an ex-jockey with no past medical history of note. The swelling was diagnosed as prepatellar bursitis by his general practitioner for which he underwent repeated aspirations of the bursa. On each occasion approximately 40 mLs of clear synovial fluid was obtained, which yielded no microbial growth.

 After six months of treatment including four aspirations and steroid injections, the patient underwent surgery to excise the prepatellar bursa from his right knee. This did not improve matters and the knee continued to swell. He was treated postoperatively with elastic compression bandages, which reduced the swelling in the knee but resulted in corresponding swelling of the lower leg. He was unsatisfied with the situation and requested a second opinion.

 The patient reported that the knee was generally uncomfortable and caused him considerable annoyance. It did not hurt and was functionally normal although made him reluctant to kneel. On examination he looked well, was wearing a compression bandage over the right knee, and walked with a normal gait. The right knee extended fully and flexed to 130°. He had an 8 × 7 cm area of fullness at the front of the right knee. Examination of the contralateral side was unremarkable.

**Figure 1:**
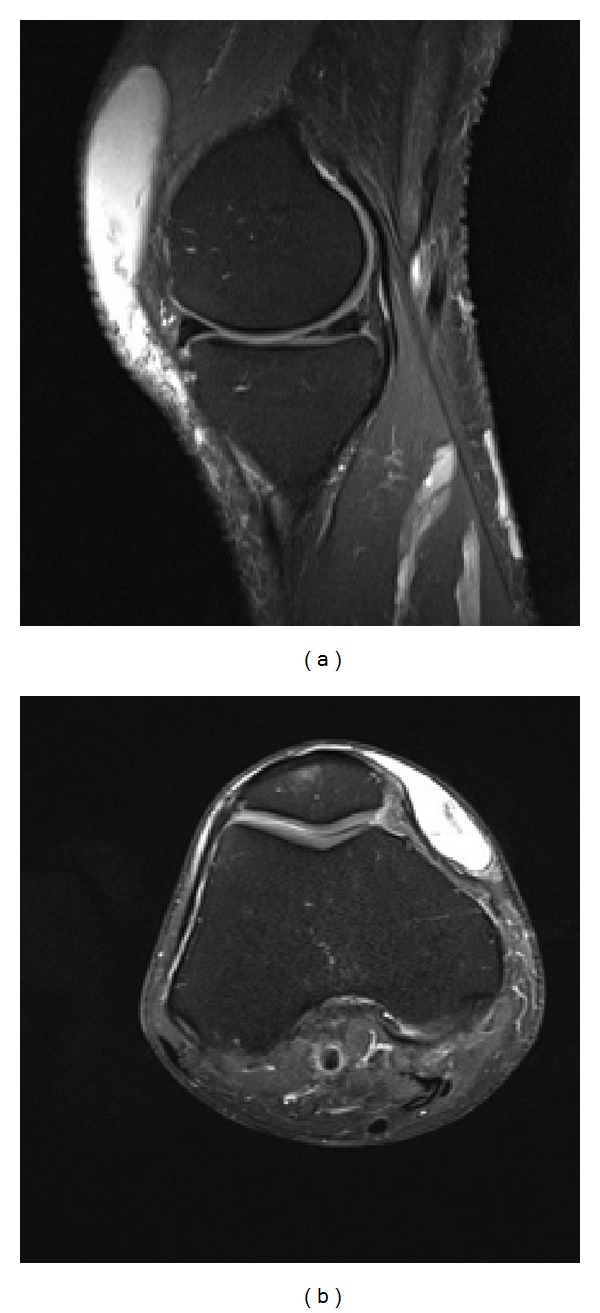
Magnetic resonance images (T2-weighted) highlighting the prepatellar collection of fluid in sagittal (a) and axial (b) sections.

A magnetic resonance image (MRI) was performed, which illustrated a large prepatellar swelling extending from the distal quadriceps expansion to the patellar tendon insertion over a craniocaudal dimension of 10 cm (Figures 1(a) and 1(b)). The rest of the MRI was normal except for an incidental finding of a free margin tear in the posterior horn of the medial meniscus.

**Figure 2:**
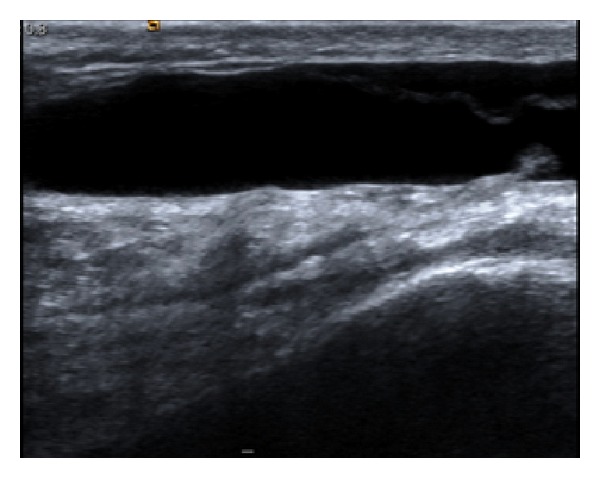
Ultrasound appearance of fluid collection lying anterior to extensor mechanism of right knee.

An ultrasound-guided aspiration was performed, which showed a large prepatellar seroma/bursa overlying the extensor mechanism (Figure 2). 18 mLs of serous fluid was aspirated and 25 mg of hydrocortisone acetate in lignocaine was subsequently injected. A pressure dressing was applied. 2 weeks later a further 22 mLs were aspirated to dryness under identical conditions and then a further 25 mLs one week later. 3 further aspirations were performed, the last of which included an injection of 6 mL of 25% dextrose (3 mL 50% dextrose in 3 mL 1% lignocaine), which was also unsuccessful.

The decision was taken to perform a joint surgical procedure with orthopaedic and plastic surgical input. The patient was prepped and draped and an incision was made through the previous bursectomy scar. The MLL was identified and decompressed with approximately 20 mLs of straw-coloured serous fluid aspirated. The dead-space was closed with a quilting suture technique: separate bites of the muscular fascia below and the superficial fascia of the skin flap above were taken using 3–0 Vicryl. This method of suture placement was to prevent shearing at the interface between the surgical planes and limit the likelihood of recurrence. 

Postoperatively he was managed in a hinged knee brace fixed in full extension for the first two weeks to allow the wounds to heal. On first clinical review, the wounds were healing well and he was able to straight leg raise and flex to 30° comfortably. At this point, the brace was adjusted to 0–30° for the next 3-4 days and then 15° more flexion every 24–48 hours. By two-month follow-up he was able to achieve a full range of movement with no suspicion of recurrence and was discharged from our clinic.

## 3. Discussion

This case illustrates an unusual cause for recurrent prepatellar bursitis resistant to conventional management. Other important differential diagnoses to exclude from MLL include nontuberculous mycobacteria [19] and acute-/chronic Gram-positive infections [20].

There have been only two previous papers in the literature addressing MLL of the knee [7, 17]. The first of these is a large series of 27 professional American Football players, and the second was a lone report following a road-traffic accident. The study by Tejwani et al. [17] represents one of the largest series of MLL and describes lesions in twenty-seven knees in twenty-four athletes between 1993 and 2006. The most common mechanism was a shearing blow on the playing surface (81%), 52% were treated successfully with compression wrap, cryotherapy, and motion exercises. 48% were treated with at least one aspiration and 22% with multiple aspirations. In only 3 cases (11%) was doxycycline sclerodesis required after three aspirations had failed to resolve the problem.

 A review of the literature using the words “Morel Lavallee” in PubMed (http://www.ncbi.nlm.nih.gov/pubmed/) returns 43 studies. Of these, 29 contain sufficient details regarding site, cause, treatment, and outcome of each lesion to be included for comparison.  Table 1 provides a detailed summary of this information with a total of 204 lesions in 195 patients. Europe has contributed the greatest number of publications on the subject. The largest series is the forementioned study by Tejwani et al. [17], followed by a series of 24 patients suffering MLL as a consequence of pelvic trauma [4]. A further sizeable series is by Neal et al. who reviewed sonographs of 21 posttraumatic fluid collections of the hip and thigh in 15 patients but unfortunately did not comment on treatment or outcome [21].

Trauma accounts for the highest proportion of causes (82%). The trochanteric region comprised 30%, thigh 20%, pelvis 18.6%, and knee 15.6% of the lesions reported. Of note, 3 cases were as a consequence of abdominoplasty [8, 9]. This was thought to result from the tangential trauma and subcutaneous dissection involved with this surgery which creates a large dead space.

 Diagnosis in most cases is made from clinical detection of a fluctuant area combined with appropriate imaging modalities. It is important to remember that the presence of a pseudocapsule may delay presentation. In one case a 56-year-old male presented with a recurrence of a large mass in his thigh, which had been excised 2 years previously after sustaining a local contusion 4 years earlier [15]. In the case of pelvic trauma, the diagnosis might only be discovered intraoperatively.

There are no clear diagnostic sonographic features of MLL which have a wide variation in appearance. As the lesion ages they become more homogenous and flat or fusiform in shape with a well-defined margin [21]. The MRI characteristics were defined by Borrero et al. [1] who reviewed images from four knee lesions sustained by young wrestlers. In each case, MRI showed a unilocular, T2 hyperintense prepatellar collection extending beyond the normal boundaries of the prepatellar bursa. These findings are also reflected in the images provided in our report. The same study concludes that although there are many imaging similarities between haemorrhagic bursitis and Morel-Lavallée effusions, making the exact diagnosis is not necessary as the management is often similar.

Management options can be categorised into operative and nonoperative. Conservative treated consists of percutaneous aspiration under ultrasound guidance with immediate postprocedure compression bandaging to prevent refilling of the space. This procedure may have to be repeated several times. Recalcitrant cases may benefit from talc or doxycycline sclerodesis. Luria et al. [13] administered talc under fluoroscopic guidance and suction drainage in four thigh MLLs. At 27-month followup all collections had gone; however, one was complicated by infection and required a second treatment. This study used five grams of sterile talc diluted in 50 mL of sterile saline.

Surgery involves evacuation of the haemolymphatic collection with excision of the pseudocapsule and debridement of necrotic tissue. The wound may be left open with or without the assistance of a vacuum dressing or closed primarily with or without a drain (Redivac) insitu. The paper by Demirel et al. [27] advocates the use of synthetic glue to close the dead-space intraoperatively. Their series of 7 thigh MLLs sustained from road traffic accidents (RTAs) all had a successful outcome when surgical drainage was combined with use of synthetic glue and compression bandaging.

The Ronceray surgical method [34] was described in a paper from Senegal by Coulibaly et al. [7]. They used this technique in 10 lesions sustained from RTA's but only reported a 40% success rate. This procedure is based on the use of aponeurotic fenestrations to allow active internal drainage and resorption by adjacent muscle fibres. The incision is centred over the collection, the entire capsule is excised and windows are cut in the aponeurosis along the entire length of the collection so that the muscle fibres communicate with the cavity. 

 Our method of quilting sutures has been described by Baroudi and Ferreira for the management of seroma formation especially following abdominoplasty [35]. It has been reported in two other papers for the management of MLLs both of which had successful outcomes [8, 15]. The report by Zecha and Missotten was for two patients who had suffered this complication after abdominoplasty. This method can therefore be considered appropriate in cases that are resistant to conservative measures. 

## 4. Conclusion

Although Morel-Lavallée lesions were described over 150 years ago, there have been only approximately 200 reports in the literature using our search method. Their presence in pelvic trauma has been associated with significantly higher surgical site infection and so their detection is of great importance. This case reflects an unusual location for this rare condition and our treatment method has only been used for three reported MLL's in the past. The diagnosis should be considered for any soft tissue swelling with a history of previous shearing trauma to that region and surgery should be reserved for recalcitrant lesions where aspiration and compression has been unsuccessful.

## Figures and Tables

**Table 1:** Summary of literature on Morel-Lavallée lesions.

Region	Publications	Aetiology	Total lesions (204)	Site	Total	Management	Total
Europe	**14** [8, 10, 12, 15, 16, 18, 22–28]	Trauma—RTA/Fall	167	Greater trochanter/hip	62	Surgical (evacuation of haematoma/necrotic tissue with debridement)	73
North America	**9** [1, 4, 6, 17, 21, 29–32]	Sport—Wrestling/American Football	32	Thigh	41	Conservative (percutaneous aspiration and compression bandaging)	47
Middle East	**2** [13, 14]	After surgery—abdominoplasty/liposuction	3	Pelvis	38	No details given (radiographic assessment only)	25
Asia	**1** [11]	Unknown	2	Knee	32	Surgical debridement with vacuum dressing	32
Australia	**1** [33]			Gluteal	13	Ronceray surgical method	10
South America	**1** [9]			Lumbo-sacral	7	Talc/doxycycline sclerodesis	7
Africa	**1** [7]			Abdominal	3	Surgical evacuation + synthetic glue	7
				Calf/lower leg	3	Quilting sutures	3
				Head	1		
				Not specified	4		
